# Genetic Diversity and Population Structure of Sardo Negro Cattle

**DOI:** 10.3390/ani16050702

**Published:** 2026-02-24

**Authors:** Blanca Catalina Colin Ibarra, Patricia Cervantes Acosta, Antonio Hernández Beltrán, Vicente Eliezer Vega Murillo, Belisario Domínguez Mancera, Vincenzo Landi

**Affiliations:** 1 Facultad de Medicina Veterinaria y Zootecnia, Universidad Veracruzana, Veracruz 91710, Mexico; bcolin@uv.mx (B.C.C.I.); pcervantes@uv.mx (P.C.A.); anhernandez@uv.mx (A.H.B.); beldominguez@uv.mx (B.D.M.); 2 Department of Veterinary Medicine, University of Bari “Aldo Moro”, 70010 Bari, Italy; vincenzo.landi@uniba.it

**Keywords:** Cebu cattle, cattle breeds, synthetic populations, pedigree

## Abstract

Livestock production in Mexico takes place in a wide range of agroecological regions, with approximately one-third of the cattle population raised under tropical conditions, where heat stress and disease pressure limit the performance of poorly adapted animals. The Mexican Sardo Negro cattle breed (*Bos indicus*) is environmentally resilient and is used for both meat and milk production; however, information regarding its population structure and reproductive management remains limited. Therefore, the genetic diversity and population structure of this breed were evaluated through pedigree analysis to support conservation strategies. Genealogical records from 8653 animals belonging to six herds located in the states of Veracruz and Chiapas, Mexico, were analyzed using ENDOG V4.8, PopRep and GRain software. The average inbreeding coefficient was 2.5%, with an increase of 0.9% per generation, a mean generational interval of 7.9 years, and a maximum pedigree depth of nine generations, although pedigree completeness was low in distant generations. The difference between the effective number of ancestors (32) and founders (37) suggests the absence of bottlenecks; however, the fact that only 21 individuals account for 50% of the genetic variability is indicative of a founder effect. Overall, the population exhibits an acceptable level of inbreeding, highlighting the importance of planned mating strategies to maintain genetic diversity and ensure the long-term conservation of the Sardo Negro breed.

## Simple Summary

In animal food production, the breeding of environmentally adapted breeds in different geographical regions is essential to maintain ecological balance and be able to respond to future challenges such as climate change, emerging diseases or resource availability. The Sardo Negro breed of cattle (*Bos indicus*) originated in Mexico and has been described as a hardy, milk and meat producing breed in tropical climates where high temperatures, humidity and pathogens have a negative impact on non-adapted animals. As a new breed with productive potential, the primary objective is to characterize its genetic diversity and population structure in different herds. To this end, a pedigree analysis was conducted using the genealogical data of 8653 animals. The animals belong to six subpopulations corresponding to different herds located in the Mexican states of Veracruz and Chiapas, where few matings between related animals were observed, as well as a low proportion of individuals with offspring. The gene flow among subpopulations also avoids genetic isolation. This information will help conserve population diversity and maintain the gene pool, phenotype, and resistance to environmental changes through artificial selection.

## 1. Introduction

The diversity of cattle species is the result of long-term human selection aimed at meeting production needs across a wide range of ecological environments. Through this process, cattle populations have been able to maintain and increase productivity while adapting to changing environmental conditions. This biodiversity provides valuable alternatives to address future challenges related to climate change, emerging diseases, human dietary requirements, and market demands [1]. Among these species, cattle (*Bos taurus*/*Bos indicus*) have successfully domesticated and now inhabit all continents, adapting to new habitats and forming populations with distinct traits shaped by cultural, environmental, and demographic influences [2]. However, sustained selection pressure exerted on livestock populations to maintain phenotype or improve productivity has increased inbreeding or mating between individuals of common ancestry, resulting in a reduction in heterozygosity and genetic variation. In some cases, these processes have resulted in reduced fertility, decreased hybrid vigor, lower disease resistance, and altered growth patterns, among other things [3,4]. Therefore, appropriate population management strategies are essential to conserve genetic diversity [5].

These general processes are particularly relevant in Mexico, where livestock production plays a significant economic and social role by providing animal protein for domestic consumption and export markets [6]. It is the seventh-largest livestock producer in the world. In terms of cattle, it is the eighth largest producer of meat and the 16th largest producer of milk [7]. The tropical region of Mexico is a major contributor to this production, where different production systems have been developed to satisfy market demand, using a wide variety of breeds and phenotypes due to the adverse environmental conditions [8]. The introduction of productive animals from different environments has often resulted in low productivity or increased production costs to express their genetic potential, so to achieve responsible and sustainable production, genetically resistant populations are needed to maintain productivity with physiological well-being and minimal external inputs to reduce the environmental impact on food production [1,9].

In this context, *Bos indicus* cattle show excellent performance and adaptability under tropical conditions, forming productive herds [10] either as pure breeds or through crossbreeding with European cattle to improve specific productive traits [11]. In this way, it is very important to preserve the diversity of locally adapted breeds, which constitute a genetic and economic resource, since they represent a germplasm that could be essential to maintain their survival percentage in the face of unexpected changes, to ensure the food supply of the population, and to preserve their cultural heritage [5,12].

Sardo Negro cattle were first registered in Mexico in 1978 by the Mexican Association of Zebu Cattle Breeders (AMCC, for its acronym in Spanish). The breed originated from crosses between *Bos indicus* cattle, imported from Brazil in the 1940s, and zebu cattle that had previously entered the country in smaller numbers via the Gulf of Mexico [13,14]. Local breeders recognized the productive potential and environmental adaptability of these animals and selectively bred them until the population was formally recognized by the AMCC, allowing its conservation and dissemination to the present day [13].

**Figure 1:**
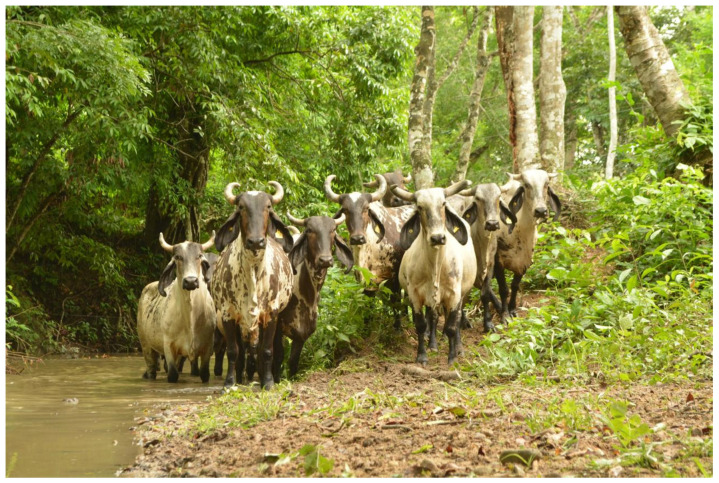
Sardo Negro breed animals, Oro Verde Ranch, México 2025. Reproduced with permission from Luis Espin.

The Sardo Negro (Figure 1) is a medium-sized, robust animal with a strong skeleton and well-defined, longitudinally distributed muscles. This breed is highly resistant to tropical climatic conditions, surpassing *Bos taurus* in this respect [15]. Breeders describe the Sardo Negro as having maternal, milk, meat, and reproductive performance compared with other *Bos indicus* breeds [16,17]. An important trait to highlight is their gentleness or docility in handling [13], a desirable trait in productive animals as it facilitates management and avoids the risk of damage to the physical integrity of the personnel handling the animals, the facilities, or the animals themselves [18].

Since its official recognition, the breed has spread to different livestock regions of Mexico, contributing to productivity within the national agroecosystem. By 2020, approximately 46,000 Sardo Negro animals had been registered by the AMCC. According to the Domestic Animal Diversity Information System (DAD-IS) [19], this breed is classified as locally adapted, with low risk of extinction and as a transboundary breed due to its recent expansion into other Latin American countries. However, due to selection during breeding, the breed may face several threats that could lead to the loss of this genetic heritage. These threats include inbreeding, which has been shown to have a negative effect on its productivity [20,21], crossbreeding with other breeds or displacement by more productive populations, and habitat loss, among other factors [22,23].

Genetic diversity within animal populations is influenced by multiple evolutionary and management-related factors. While migration and mutation can increase genetic diversity [24], bottlenecks and high levels of inbreeding can substantially reduce it [25]. Therefore, understanding the genetic relationships among individuals is essential for maintaining diversity when designing breeding programs or implementing conservation measures [26]. Although molecular tools are currently available to assess genetic diversity [27], genealogical records remain a valid and informative approach for evaluating population structure, inbreeding and genetic diversity, particularly in managed livestock populations [5,28].

Despite extensive studies on several zebu breeds such as Brahman, Nelore and Gyr whose genetic diversity and population structure are well documented [29], information for the Sardo Negro breed remains limited and fragmented. This breed differs from cosmopolitan zebu populations due to its relatively recent origin, smaller population size, and localized management under predominantly extensive production systems. These distinguishing characteristics suggest population dynamics that cannot be directly extrapolated from those of better-studied breeds. Consequently, the lack of breed-specific genetic information highlights the need for targeted studies to support effective breeding and conservation strategies for the Sardo Negro breed. Due to imminent climate change, livestock breeding strategies must now focus on the conservation, use, and genetic improvement of locally adapted breeds to enhance their resilience [30].

Due to the reduced population size and the recurrent use of a limited number of breeding animals, the Sardo Negro breed may be potentially exposed to an increase in inbreeding and a loss of genetic variability, phenomena that have been described in bovine populations with similar characteristics. In this context, pedigree analysis constitutes a fundamental tool for evaluating the genetic structure of the population and for guiding reproductive management and conservation strategies.

Accordingly, this study aims to update and complement recent genomic evaluations by conducting a comprehensive pedigree analysis to quantify genetic variability and population structure in the Sardo Negro breed [28,31]. The results are expected to contribute to the development of active conservation strategies that enable effective genetic management during breeding, ensuring that genetic progress does not compromise the breed’s defining characteristics. Furthermore, this research will promote knowledge of the breed in areas of productive interest, such as milk and meat production, thereby supporting its strategic inclusion in sustainable production systems and demonstrating that conservation and productivity are complementary objectives in the management of local zoogenetic resources.

## 2. Materials and Methods

Genealogical information from 8653 Sardo Negro cattle born between 1985 and 2022 was used for this study. Animals were selected using a convenience sampling approach based on the availability and completeness of pedigree records provided by collaborating breeders. Although this sample does not include the entire population, it encompasses the actively managed breeding nucleus across multiple herds and generations, making it relevant for assessing genetic structure and inbreeding dynamics. The analyzed dataset represents approximately 18% of the animals registered up to 2022. The animals belonged to six different herds (subpopulations), members of the AMCC. These herds are representative of each region in which this breed of cattle is raised.

**Figure 2:**
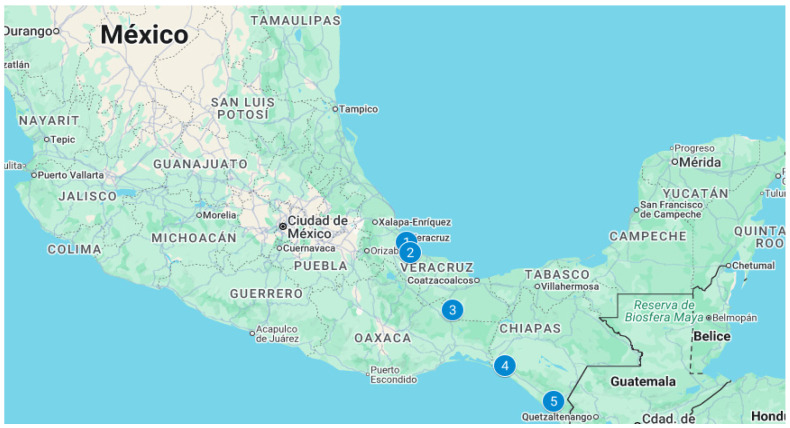
Location of the herds on the Mexican map. Map data ©2025 INEGI, Google.

The herds can be found in the tropical region of Mexico, with a warm sub-humid climate and summer rains. In the state of Veracruz, Gulf of Mexico region, in the municipalities of Medellín (temperature 24–28 °C, rainfall 1900–2600 mm; subpopulation 1) and Tlalixcoyan (temperature 24–28 °C, rainfall 1400–1600 mm, subpopulation 2), Jesús Carranza (temperature 24–26 °C, rainfall 1200–2500 mm; subpopulations 3 and 4). In southeastern Mexico, in the state of Chiapas, the municipalities of Tonalá (temperature 14–30 °C, rainfall 1200–3500 mm; subpopulation 5) and Villa de Comatitlán (temperature 22–30 °C, rainfall 1500–4000 mm; subpopulation 6) [32]. According to Köppen’s climate classification system, modified by García in 1988 to adapt it to the Mexican environment, subpopulations 3 and 4 are classified as climate Aw_2_, subpopulations 1 and 2 as climate Am and subpopulations 5 and 6 as Aw_2_ (w) [33] (Figure 2). These climatic conditions impose significant thermal stress and disease challenges, thereby increasing selective pressure in favor of environmentally adapted breeds.

Each herd provided data on all their registered animals, including individual identification, sire and dam identification, date of birth, sex, and subpopulation to which they belong. Information on individuals who have left the herd without registration is not included. As is common in genealogical databases, incomplete parental information was present, particularly in early generations, which may influence the depth of pedigree reconstruction and lead to underestimation of inbreeding coefficients.

Prior to analysis, pedigree data were edited to identify and correct inconsistencies, duplicated records, and missing identifiers. To this end, it was verified that parents were born before their offspring and that the registered sex matched the parental role (sire/dam). It should be noted that not all individuals born in these herds receive the AMCC record for various reasons (phenotype, commercial destination, disease, death, etc.), so data from entire families are not included. Animals with unknown parents were retained, as their inclusion is essential for estimating founder contributions and pedigree completeness.

Demographic characterization of the population was performed using POPREP v1.0 software (Neustadt, Germany) [34]. This information could be used to detect the unequal use of breeding animals.

Pedigree quality was assessed by estimating pedigree completeness, the percentage of known ancestors per generation and the completeness index (CI) [35]; in addition, the number of complete generations traced was estimated for each individual, which separates the offspring from its most distant generation in which two ancestors are known; the maximum number of generations traced as the number of generations separating the individual from its most distant ancestor and complete equivalent generations as the calculated sum of each known ancestor [36]. These indicators were calculated using the software ENDOG v4.8 (Madrid, Spain). They supported the interpretation of inbreeding and relatedness estimates.

ENDOG v4.8 [36] was also used to estimate the generation interval and genetic diversity parameters: inbreeding, relatedness, effective population size, ancestry explaining genetic variability, individual genetic conservation index and contribution of subpopulations to total diversity.

The generation interval (GI) was calculated as the unit of time it takes to replace a set of parents. The mean age of breeding males and females at the time of birth of their reproductive or non-reproductive offspring (AB) was also calculated [37]. These parameters were analyzed in their four selection paths: sire-son, sire-daughter, dam-son, dam-daughter standard error was calculated by the analytic method. This research does not consider the exclusion of animals after genealogical analysis, so quality control based on generational intervals or traced generations was not applied.

The inbreeding coefficient (F), defined as the probability that two gametes forming an individual carry identical alleles by descent (IBD), resulting from mating between individuals with a common ancestor or relatives [38], was calculated for all pedigree members according to established equations [39]. The average relatedness coefficient (AR), defined as the probability that a randomly selected allele in the study population belongs to a particular animal, thus indicating its relationship to the herd, independent of pedigree. Both were calculated for each individual and in each estimated generation. These parameters provide a quantitative measure of relatedness within the population. It is essential for assessing genetic risk, interpreting population structure, and designing effective breeding and conservation strategies.

The effective population size (Ne) is the number of parents that would result in the actual increase in inbreeding if they contributed equally to the next generation. It is calculated using the formula Ne = 1/(2∆F_OBS_), where ∆F_OBS_ is the observed increase in inbreeding per generation. This is used to compare the efficiency of natural selection, artificial selection, and migration processes (ΔF = Ft − F (t − 1)/1 − F (t − 1)). This metric tells us the size of an ideal population that loses heterozygosity at the same rate as the primary population of interest and is always smaller than the real size [37,38]. However, this model may not adequately adjust for the size and depth of the pedigree, leading to an overestimation, so we also consider calculating Ne from the decision tree constructed by Groeneveld et al. (2009), which compares different estimation methods and leads to the selection of the best method through its completeness and stability [34].

GRain v2.2 software (Vienna, Austria) was used to calculate the ancestral inbreeding coefficient [40]. The coefficients of classical inbreeding, ancestral inbreeding (Fa_BAL_ and Fa_KAL_) and the coefficient of ancestral history (A_HC_) were calculated using a stochastic method (gene dropping) with 10,000 replicates. Fa_BAL_ is defined as the probability that each allele of an individual has been identical by descent at least once in previous generations. Fa_KAL_ represents the part of the genome in which the alleles are currently in an autozygous state and were also present at least once in an ancestor of the animal. A_HC_ is the number of times during pedigree segregation that a randomly selected allele is identical by descent [40]. These parameters were calculated to characterize the accumulation of inbreeding over time and to distinguish between recent and historical inbreeding.

The probability of origin of genes in the study population was calculated with the ancestries that explain the genetic variability: effective number of founders (ƒe) as the number of ancestors with unknown progenitors of equal contribution that are expected to generate the same genetic diversity as in the population under study, expressed as a fraction of the genes contributed by the founders and maintained in the population; effective number of ancestors (ƒa), founders or not, this is the minimum necessary to explain the complete genetic diversity of a population. The difference between ƒe and ƒa explains the presence of bottlenecks or the reduction in its members in a period, with a consequent change in allele frequencies as the population evolves [38].

In order to identify the animals with the highest contribution to conservation and to maintain the full range of alleles in the population, the Genetic Conservation Index (GCI) was calculated for each individual in the analyzed population, which identifies the genetic contribution of the founders and shows the degree of individual conservation from the base population [41].

Wright’s F-statistics were also estimated to assess genetic diversity due to inbreeding. They indicate the degree to which heterozygosity in the population has been reduced from the identity by descent in the pedigree analysis. FIS was calculated as the inbreeding coefficient of an individual with respect to its subpopulation, indicating the reduction in random mating, and is defined as the probability that two alleles in an individual are identical by descent with respect to the subpopulation. FST refers to the correlation of alleles in different individuals of the same population and was calculated from the average inbreeding of a subpopulation relative to the total population; it is the expected inbreeding from random mating. Finally, FIT indicates whether two alleles of the same locus in an individual are IBD relative to the metapopulation, calculated as the inbreeding coefficient of an individual relative to the total population [37,38].

Finally, the genetic significance of herds was calculated using the contribution of breeding males to determine whether subpopulations were isolated [42]. Subpopulations are identified as nucleus herds if breeding animals use only their own males, multiplier herds if they use external males and sell their own, and commercial herds if they accept males from other herds and never sell their own. This assessment provides information on gene flow, identifies unequal parental contributions, and supports the design of breeding strategies aimed at maintaining genetic diversity and reducing inbreeding.

## 3. Results

Genealogical data were obtained for 8653 animals registered from 1985 to 2022 from the six subpopulations that made up the present study. Each subpopulation contributed from 1 to 6: 2817, 176, 1002, 430, 3872 and 356 animals, respectively.

### 3.1. Population Structure in Sardo Negro Cattle

#### 3.1.1. Population

Table 1 summarizes the demographic characteristics of the study population. The population consisted of 56% females and 44% males. Only 30% of the individuals are breeding animals. Among females, 49% were recorded as dams, whereas only 6% of males had registered offspring. The average number of offspring was three per cow and 31 per bull. Overall, 70% of the individuals had no registered progeny. Likewise, the reference population is 79%, i.e., with both parents known, 20% with both parents unknown, and 0.8% with at least one parent known.

**Figure 3:**
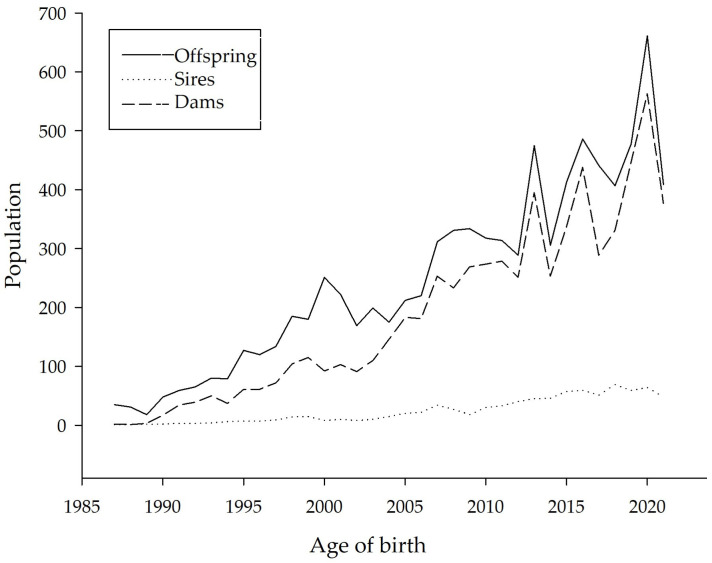
Number of descendants per year of birth and number of dams and sires who bore them.

Figure 3 shows the annual number of offspring born, together with the number of sires and dams involved in their production. The data shows a progressive increase in population size over time. During the first ten years of the study period, fewer than 200 calves were born per year. From 2000 onwards, annual births increased to between 200 and 400 calves per year, reaching up to 600 calves per year during the most recent decade. The number of dams is related to the number of calves born, whereas the number of sires used for breeding remained comparatively low throughout the study period and showed little variation over time.

#### 3.1.2. Pedigree Completeness

**Figure 4:**
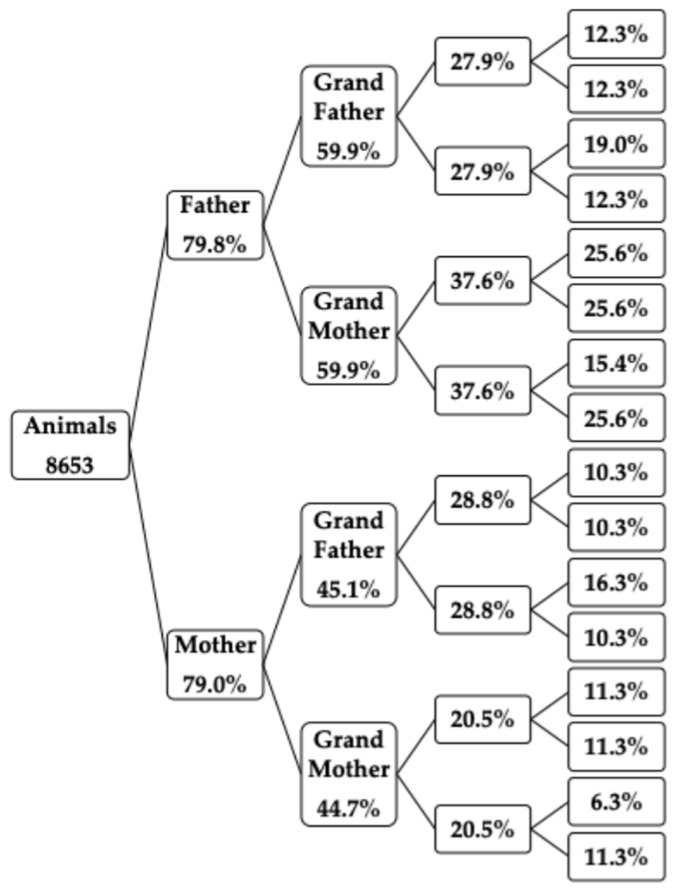
Percentage of known ancestors.

Figure 4 shows the percentage of known ancestors by generation. Approximately 80% of the animals had known parents. However, this percentage decreases in distant generations: 60% of paternal grandparents, 45% of maternal grandparents, 28% of paternal great-grandparents, and 20% of maternal great-grandparents are known. The percentage decreases further in older generations. Across the generations, a higher proportion of known ancestors was observed on the sire line compared with the dam line.

**Figure 5:**
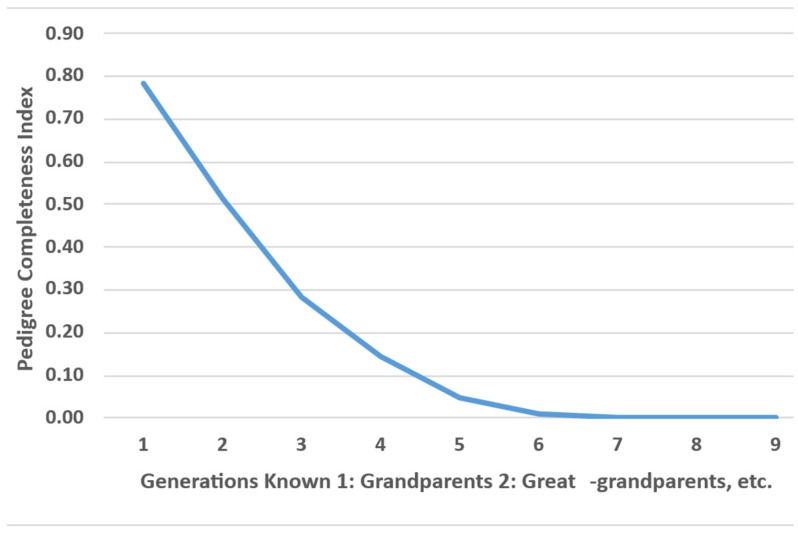
Pedigree Completeness Index (CI).

Figure 5 represents the pedigree completeness index (CI) across generations. The highest CI values were observed in the most recent generations, followed by a marked decline in older generations.

The pedigree depth of the population was characterized by an average and maximum values of 2.8 and 9 maximum traced generations (MG), 1.2 and 4 complete generations (CG), and 1.8 and 5.2 equivalent generations (EG), respectively. These values indicated a limited number of recorded generations. Additionally, an increase in inbreeding was observed across pedigree depth measures, with rates of 0.89% per MG, 3.12% per CG and 2.08% per EG.

#### 3.1.3. Generational Interval

The average generation interval (GI) was 7.9 years and the average age of the parents at the birth of their offspring (AB) was 8.2 years, a slightly higher value when the reproductive status of the offspring is not considered. Trends across the four selection routes are presented in Table 2, where no statistically significant differences were observed between the different breeding routes.

### 3.2. Inbreeding and Diversity in Sardo Negro Cattle

#### 3.2.1. Inbreeding and Relatedness

**Figure 6:**
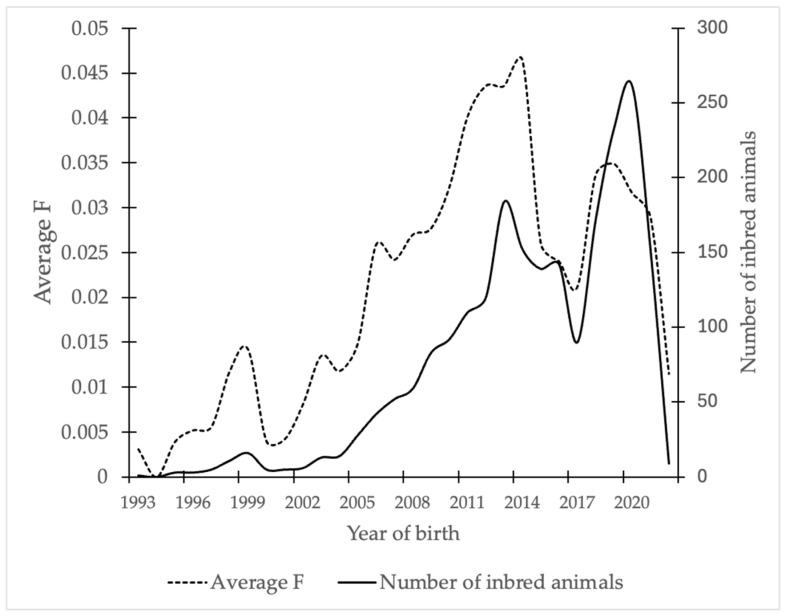
Inbreeding coefficient (F) and number of inbred animals in Sardo Negro cattle, by year of birth.

Table 3 presents the average of the inbreeding coefficient (F), average relatedness coefficient (AR), and effective population size (Ne) by the maximum generation of the individuals making up the population. The average F calculated for each subject of the analyzed pedigrees was 2.5%, with values ranging from 0 to 5.2% across MG. As shown in Figure 6, the average inbreeding by year of birth increased over time. A similar trend was observed in the proportion of inbred animals within the population.

When inbred animals were analyzed by generation (Table 3), generations 2 and 3 exhibited mean F of 15.7 and 13.2%, respectively. However, because these generations include relatively few individuals (9.5% and 28.4% of the population, respectively), this is not reflected in the overall generational average. Similarly, in more distant generations, where the proportion of inbred individuals is higher (up to 75%), animals exhibit a lower average F value (4.0%). Mating events contributing to inbreeding included matings between full siblings (0.08%), half-siblings (5.2%), and parents-offspring pairs (1.5%).

When inbreeding was evaluated according to the number of CG (four complete generations), values of 14.9% were obtained in the most distant generation (fourth), followed by 8.3% in the third generation, 5.6% in the second, 0.5% in the first and 0.0% in the most recent generation.

Differences in inbreeding levels were also observed among herds included in the study. Herds 1 to 6 reported inbreeding coefficients of 3.2%, 1.2%, 1.3%, 1.8%, 2.5% and 2.6%, respectively. Herds with a larger number of animals report higher levels of inbreeding.

The average relatedness coefficient (AR) for the population was 2.5%. Values of MG ranged from 0.1 to 4.2, with higher values in earlier generations and lower values in more recent generations.

According to the classical inbreeding coefficient estimated using the gene dropping method, the average was 2.6%. The averages of fa_BAL,_ Fa_KAL,_ and A_HC_ were 2.7%, 2.9% 0.006%, respectively. Overall, 15% of the individuals were inbred, 46% of them showed no evidence of ancestral inbreeding. In contrast, 17% of the non-inbred animals exhibited some degree of ancestral inbreeding, so they have previously been exposed to inbreeding by their ancestors, but this inbreeding is low. According to the A_HC_, values ranged from 0 to 4. Individuals with an ancestral inbreeding history had an average A_HC_ value of 1.2, indicating that these animals have been exposed to ancestral inbreeding at least once with low frequency (5% of the population).

#### 3.2.2. Effective Population Size

The Ne of the population was estimated as 57.6 based on the MG, and as 22.1 when calculated using the individual increase in inbreeding. It is a low value compared to the census. When Ne was evaluated across generations (Table 3), a value of 124 was observed in the seventh generation, increasing to 387 in the fifth generation, and subsequently decreasing to 22 in the most recent generations. In some generations, negative Ne estimates were obtained and are indicated with a minus sign (−) because the model is poorly fitted due to artefactual conditions, derived from a wide generational interval, the overlap of generations, and the unbalanced ratio between males and females.

Ne was calculated by different methods and subjected to the decision tree; it was found that the method with the highest applicability is the one that identifies the complete ancestors born in that generation. The fitness of the model is assessed in terms of its stability and completeness. The Ne obtained by this method supports the Ne of 22.

**Figure 7:**
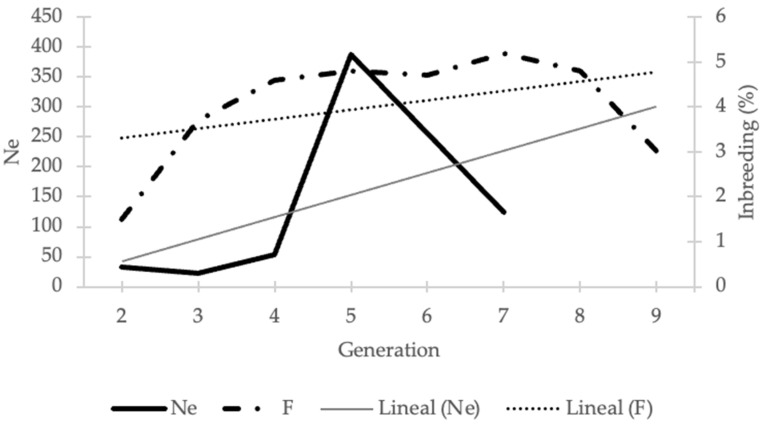
Trends in inbreeding and Ne in Sardo Negro cattle, by generation.

Figure 7 shows the temporal trends of F and Ne within the study population. The results show a marked decline of Ne in recent generations, while changes in inbreeding levels were comparatively smaller over the same period.

#### 3.2.3. Ancestors

The population consisted of 8653 individuals, of which 6838 animals with both parents known comprised the reference population (Table 4). This reference population originated from the contribution of 1175 founders, representing 13.6% of the total population. Additionally, a base population of 1815 individuals (21.1%) had one or both parents unknown. The ƒe was estimated at 37, while ƒa was 32. Only 21 ancestors explain 50% of the genetic variability of the study population.

#### 3.2.4. Genetic Conservation Index

**Figure 8:**
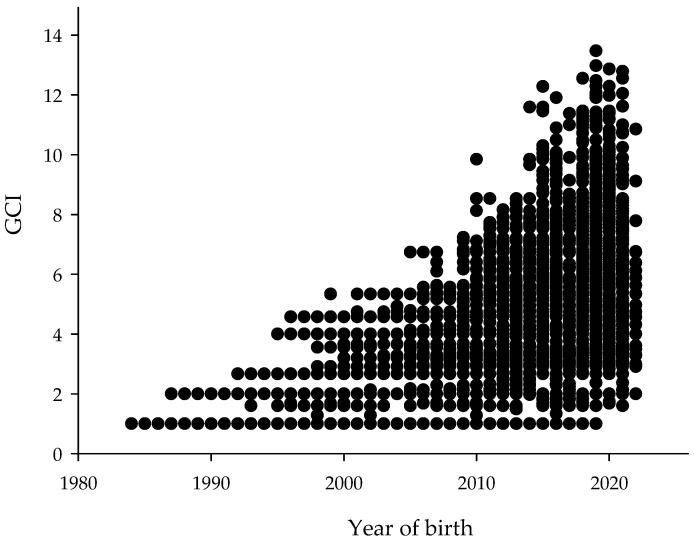
Distribution of Genetic Conservation Index (GCI) by year of birth in Sardo Negro cattle.

The GCI calculated for each subject ranged from 1 to 13.47. The mean GCI for the entire population was 3.3. As Figure 8 shows, individuals born in recent years exhibited GCI values spanning the full observed range.

#### 3.2.5. Genetic Differentiation Between Subpopulations

Wright´s F-statistics were estimated to assess genetic differentiation among subpopulations; the F_ST_ value was 0.018. The F_IS_ value was 0.005 and the F_IT_ value was 0.013.

Male genetic contributions differed among herds. Five of the six subpopulations were classified as multiplier herds, as they use external males (33%, as the remaining percentage of the average number of own males in the first five herds classified as multipliers) and sell their own males as sires. One subpopulation was classified as commercial using external bulls (39%, as the difference in the percentage of the herds’ males in the only commercial herd), but does not share its males for breeding with other herds.

Most herds use the highest proportion of their own sires, up to 75%. However, the proportion of progeny from other herds is low, ranging from 0.6 to 8.2% in multiplier herds (Table 5).

## 4. Discussion

### 4.1. Population Structure

In this Sardo Negro population, which has been increasing in size each year and is subject to breeder selection, proper mating management is essential to preserve the genetic diversity that has enabled the breed to adapt to its environment and to ensure its long-term sustainability under future changes. To this end, breeders have implemented genealogical tracking, artificial insemination, and the introduction of external bulls as strategies to improve the breed. The Sardo Negro breed in Mexico has a sire-to-female ratio close to 50%, with an average of three progeny per cow and 31 per bull. Pedigree records show significant population growth over time, although pedigree completeness remains low in older generations.

The female-to-male ratio in this Sardo Negro population is similar to that reported for other cattle populations, such as Gyr and Sindi cattle, and to other zebu breeds in Brazil, where values close to 50% have been observed [29]. A comparable pattern has also been reported in artificial insemination pregnancies [43], although natural mating has been shown to favor the higher proportion of female offspring [44]. This proportion may also be influenced by the selection criteria used for AMCC registration, as animals that do not meet desirable phenotypic or health standards are excluded from the pedigree. However, reproductive opportunities are not equally distributed among animals. A relatively small proportion of males contribute offspring compared to females, as reported in several meat-producing cattle breeds in Mexico (*Bos taurus* and *Bos taurus*/*Bos indicus*) [45], This pattern reflects common breeding practices, in which most females are retained in the herd, while only a limited number of outstanding males are kept, as maintaining few males is more economically profitable [46]. Other males are sold as sires to different herds or used for commercial crossbreeding for meat or milk production and are therefore not registered in the breeders’ association [47]. This confirms that the population has been subject to moderate artificial selection, characterized by a reduced number of breeding males and high selection intensity in the male population, which limits the genetic diversity transmitted to subsequent generations.

Unequal reproductive contribution between sexes is also observed in wild populations. For example, in deer populations, males establish dominance hierarchies and compete for females, resulting in a limited number of males producing offspring, with older and heavier individuals having a reproductive advantage [48]. A similar pattern has been reported in Berrenda cattle, where the average number of offspring per cow and bull was 3 and 36, respectively [49]. Although this breed is found in the Iberian Peninsula, it has been classified as an endangered native breed that contributes to the ecological balance of the Dehesa ecosystem. Therefore, the number of breeding animals directly influences the genetic structure of future generation and the rate of genetic progress depends on the age of breeding animals and their replacement rate [37]. These factors must be carefully considered when improving genetic performance while preserving diversity. As mentioned above, these parameters may also be influenced by the proportion of animals registered in the AMCC.

Changes in livestock population are determined by an interaction between genetic [50], environmental [51], management [52] and market-related factors [53]. For example, the population of the Negra Andaluza breed has declined mainly due to changes in management practices and the introduction of foreign meat-producing breeds [54]. In contrast, several *Bos taurus* populations have experienced demographic growth and have colonized new geographical areas. However, this does not guarantee long-term success, as the loss of genetic diversity can threaten population viability [55,56]. These results emphasize that population growth alone is insufficient to ensure genetic sustainability and that mating strategies must be guided by pedigree-based parameters to control inbreeding accumulation. Therefore, the population increase observed in the Sardo Negro breed must be managed carefully to avoid the negative consequences of artificial selection, as occurred in the Belgian Campine breed, where preserved genetic diversity facilitated its reintroduction [57].

In general, the results of the analysis are more accurate when pedigree quality is high and includes nine or more known generations [5]. For this reason, a long, complete, and reliable genealogical record is therefore essential [58]. The relatively low quality observed in this study is likely related to the recent establishment of the breed. A marked contrast was observed between the overall average inbreeding coefficient (F) of 2.5% and the average F based on complete generations (CG) of 14.9%. The contrast between these estimates underscores the importance of pedigree depth and accuracy when interpreting genetic parameters.

In Mexican meat-producing cattle populations, including *Bos taurus* and *Bos taurus*/*Bos indicus* crosses, the proportion of known ancestors ranges from 67% to 93% in the first generation, from 49% to 92% in the grandparent generation, and from 38% to 87% in the great-grandparent generation [45]. The main difference is between breeds: those with a longer registration time show a higher percentage of known ancestors. Consistent with the present study, a slightly higher percentage of known ancestry has been observed in the paternal line, likely due to the limited number of carefully selected sires with documented ancestry. Similar patterns have been reported in Romosinuano cattle populations in Mexico, where the CI reaches its lowest value in the sixth generation and increases to 0.8 in more recent generations [59]. In contrast, *Bos taurus* Holstein cattle, with approximately 60 years of records, show CI values above 0.8 in the most recent half of the pedigree [60]. Consequently, the low pedigree depth observed in this study (EG = 1.8) contrasts with that reported in other cattle populations, where higher pedigree depth and CI values correspond to EG values ranging from 4.8 in Sindi cattle to 7.2 in Brahman cattle [29]. The limited number of recorded generations is consistent with the relatively recent origin of the Sardo Negro breed.

The GI observed in the Sardo Negro population is intermediate compared with other *Bos indicus* breeds and higher than that reported for *Bos taurus* cattle, with no distinction between sires and dams. This may reflect the adaptation of zebu cattle to tropical environments, where resilient breeds are required and where physiological characteristics lead to a later onset of reproductive life and longer herd retention compared with European breeds [61]. A previous study on Sardo Negro cattle reported an average GI of 7.5 years [28]. Among Brazilian zebu breeds, the lowest GI has been reported for Brahman cattle (6.9 years) and the highest for Indubrasil cattle (9.8 years) [29], with the longest intervals observed in male progeny. Differences in GI between sexes and breeds can be explained by variation in replacement age, number of offspring, and herd-specific reproductive management practices.

The absence of differences between sires and dams in the present study may be explained by the prolonged use of certain breeding animals in both sexes. In Romosinuano Creole cattle, GI values range from 6.5 to 7.2 years [59] while in Berrendo cattle they range from 4.9 to 7.3 years, with higher values observed in females due to their longer reproductive lifespan [49]. In contrast, selection aimed at improving economically important traits has reduced GI in Limousine and Charolais cattle [62], as well as in Mexican beef breeds, where GI ranges from 5.1 to 6.5 years. Nevertheless, local European breeds with limited reproductive management may exhibit longer GI values (8–13 years) than those observed in the present study, whereas cosmopolitan breeds included in artificial insemination programs typically show shorter GI values (6–7 years) [5]. Thus, the adoption of reproductive technologies may be a key factor contributing to reduced GI. Reducing GI allows for faster genetic improvement, whereas maintaining a longer GI can help slow the rate of genetic diversity loss over time [63]. The wide GI observed in this population is considered advantageous because it can prevent rapid loss of genetic diversity. Therefore, breeding programs must balance genetic progress with diversity conservation by implementing mating strategies that minimize genetic erosion, even when shorter GI values are desired.

### 4.2. Inbreeding and Diversity

Inbreeding (F), resulting from the systematic selection of related breeding animals to maintain specific phenotypic characteristics, can vary widely among domestic animal populations. One of the primary factors contributing to inbreeding is small population size [64]. The Sardo Negro breed originated from a limited number of animals selected for superior phenotype from Brazil and crossed with Creole Zebu cattle. This founding structure may have resulted in a high frequency of related matings during the early stages of breed formation; however, the lack of historical records prevents direct verification, as reflected by the CI values in the most distant generations. The average inbreeding coefficient observed in the present population is considered acceptable, as it remains below 6.25% [65,66]. A previous study reported an average F of 3.6% for the Sardo Negro breed [28].

In Brazil, average F values ranging from 1.9 to 6.2% have been reported in different zebu breeds [29] while values between 2.4 and 12.9% have been observed in fighting cattle herds in Mexico [67], both of which are adapted under harsh environmental conditions. In fighting cattle, high inbreeding levels are primarily attributed to the small number of founder animals. Local European breeds reported by Fabbri et al. (2019) show F values between 1.2% and 7.3% [5]. Although these breeds exhibit low frequencies of sibling and half-sibling matings, higher inbreeding values are associated with parent–offspring matings, reflecting limited reproductive control typical of local breeds [5]. In the present population, the low frequency of sibling and parent–offspring matings, combined with a higher proportion of half-sibling matings, suggests directed mating strategies aimed at fixing desirable phenotypes.

In American Angus cattle, an average F of 5.9% has been reported, along with evidence of inbreeding depression affecting growth traits [4]. Similarly, although Holstein cattle in Mexico show an average F of 2.6% [21], individuals with F values greater than 5% exhibit reduced milk, fat, and protein production. Given that these populations are managed under conditions similar to those of the Sardo Negro breed, characterized by limited numbers of breeding herds and sires, it is essential to monitor and control inbreeding levels to prevent inbreeding depression. In general, inbreeding levels up to 6.25% are considered acceptable, whereas values above 12.5% are regarded as critical [65,66]. The inbreeding coefficient in the present population remains within acceptable limits, implying a relatively low loss of heterozygosity, and can be reduced further through appropriate breeding management.

The contrast between the overall inbreeding coefficient (F) and those derived from CG reinforces the likelihood of underestimation due to incomplete pedigree data. As pedigree completeness improves, higher inbreeding levels become detectable. Therefore, future population management should be based on complete and accurate data to prevent a rapid increase in inbreeding.

The ancestral inbreeding coefficients observed in this study (Fa_BAL_ = 0.027 and Fa_KAL_ = 0.029) are comparable to values reported in other cattle populations, although their differences reflect distinct selection patterns. Hinrichs et al. (2015) [68] reported a higher Fa_BAL_ value (0.082), indicating a higher probability that each allele has been IBD at least once in previous generations, and a lower Fa_KAL_ value (0.007) has a lower probability of being IBD both at present and in its ancestors, so the inbreeding could have originated in more distant generations. In that study, approximately 26% of non-inbred animals exhibited ancestral inbreeding, a higher proportion than observed here (17%), likely reflecting stronger and longer-term selection intensity in dairy cattle. In contrast, Wirth et al. (2023) reported Fa_KAL_ and AHC values of 0.002 and 0.012, respectively, in German Brown cattle with an average F of 2.3%, suggesting more recent selection and inbreeding processes [69].

These studies reported higher pedigree depths (GE = 4.8 and 6.2, respectively) than observed in the present study (GE = 1.8), which may contribute to more accurate parameter estimates. Moreover, these authors documented significant inbreeding depression in productive traits [69], potentially limiting population viability. Because inbreeding leads to the expression of deleterious alleles and represents a considerable threat to population survival [70], it is essential to mitigate its negative effects when inbreeding is combined with selection [71]. Selection strategies should therefore aim to eliminate deleterious alleles while minimizing the loss of genetic diversity. Given the population structure of the Sardo Negro breed, such strategies could help prevent inbreeding depression.

The average relatedness value of 2.5% observed in this study indicates a high proportion of individuals sharing alleles within the population, increasing the risk of inbreeding if reproduction is not adequately controlled [72]. Consequently, breeding programs should prioritize animals with average relatedness (AR) values below 1. Fabbri et al. (2019) demonstrated that higher inbreeding levels are associated with a greater number of relatives, particularly in local breeds with limited reproductive management [5]. Neglecting population relatedness may therefore compromise genetic diversity in future generations.

According to FAO recommendations, a Ne of at least 50 animals is required to avoid genetic diversity loss [1], Although Ne exceeded this threshold in earlier generations, a value of 20 was reported for the most recent generation, despite continuous population growth. This trend may increase the risk of genetic variability loss. In Italian local breeds, Ne values range from 14 to 40, whereas cosmopolitan breeds in the same country show Ne values between 90 and 133 in two different populations [5]. In Mexico, different cosmopolitan breeds dedicated to meat production report Ne values ranging from 24 to 155, although being transboundary breeds, it is possible to induce the flow of genetic material to avoid loss of diversity [45]. Overall, variation in Ne among cattle breeds reflects differences in breeding strategies and genetic history. Under these conditions, reductions in heterozygosity are more likely than losses of allelic diversity [37]. It should be noted that the present study includes only a subset of the Sardo Negro population in Mexico, and the total Ne for the country is likely higher [28]. Although the census population is large, the low Ne reflects unequal reproductive contributions between sexes and among families, a pattern typical of cattle populations managed using controlled mating and artificial insemination. Under these conditions, reductions in heterozygosity are more likely than losses of allelic diversity.

The ƒe/ƒa ratio observed in this study is similar to that reported by Fabbri et al. [5], with values between 1.1 and 1.2 for local breeds, indicating no evidence of a recent bottleneck. In contrast, cosmopolitan Charolais and Limousine breeds in Italy show ƒe/ƒa values between 2 and 3, reflecting bottlenecks primarily associated with intense selection, as also reported in Mexican Charolais cattle [73]. However, a founder effect may still be present in the Sardo Negro population, as a limited number of animals account for 50% of the genetic contribution, a pattern also observed in the Italian Tuscan breed [5]. Although Sardo Negro cattle are subject to selection, the results suggest that irreversible changes in population structure have not yet occurred. However, failure to increase effective population size and to balance the use of breeding animals may lead to future losses of diversity or heterozygosity.

Overall, average levels of inbreeding and relatedness are relatively low, considering that this population is derived from controlled mating. However, due to low pedigree completeness, some of these indicators may be underestimated. In addition, conditions that could lead to a loss of genetic diversity have been identified, such as the presence of highly inbred individuals, a low effective population size, and a founder effect. According to the evaluated parameters, genetic diversity is still preserved, but the low pedigree integrity may obscure the true situation, highlighting the need to approach conservation as an ongoing process requiring active management.

### 4.3. Implications for Conservation

The GCI values obtained in this study are comparable to those reported by Figueredo et al. (2019) for Brazilian Somali sheep [74]. These authors suggested that animals with higher GCI values, reflecting greater founder contributions, can be strategically used in mating programs to preserve founder alleles. Accordingly, targeted mating strategies involving animals with high GCI values should be implemented, and each subpopulation should identify individuals with these characteristics. In contrast, Murciano Granadina goats exhibit a low average GCI (1.64), where unbalanced founder contributions hinder the conservation of the breed’s original genetic diversity [75]. Then, higher GCI values indicate greater founder contributions, and selective matings involving individuals with high GCI can help preserve founder alleles. However, at the time of data collection, this indicator was not considered in mating decisions.

Based on the Wright’s statistics obtained in the present study (FIS 0.005, FST 0.01, FIT 0.013), the Sardo Negro population shows a level of genetic differentiation comparable to that reported in Mexican fighting cattle populations, where an average FST of 0.0086 was observed, although with a lower FIS value (−0.167), suggesting an increase in heterozygotes [76]. Ribeiro et al. (2021) reported FIS, FST, and FIT values of −0.157, 0.104, and –0.036, respectively, in another cattle population, indicating balanced levels of homozygosity and heterozygosity despite inbreeding among subpopulations [77]. Together, these results indicate an intermediate level of population differentiation, consistent with previous genome-wide analyses of the Sardo Negro breed [31]. This genetic structure provides substantial variability, enhancing the breed’s potential for long-term survival.

These findings are further supported by herd-based population structure analyses, which show that most herds act as multipliers with genetic exchange among subpopulations, a pattern commonly observed worldwide, particularly with the use of assisted reproductive technologies. Although the exchange of animals among herds is recommended to prevent genetic diversity loss [63], such exchanges must be carefully managed to ensure diversity conservation [78]. This requires detailed knowledge of the genetic background of breeding animals to avoid unintended increases in inbreeding.

According to FAO, Latin American and Caribbean countries possess high levels of national biodiversity but have relatively few active conservation programs [1]. As with other breeds in the region, the ancestors of the Sardo Negro breed were imported and subsequently improved through breeding programs to produce purebred animals adapted to local conditions. Therefore, preserving their genetic diversity is essential. The results of this study indicate that maintaining genetic diversity in Sardo Negro cattle requires careful management of mating strategies, taking into account Ne, F, and GCI values, particularly given the current population growth (Figure 3). Moreover, zebu cattle play a critical role in ensuring food security in tropical regions.

Overall, the findings demonstrate that conservation measures are necessary to prevent irreversible loss of genetic resources, which constitute valuable regional assets essential for biodiversity conservation, food production, and resilience to future challenges [79].

Based on the parameters estimated in this study, several strategies can be proposed to ensure the long-term genetic sustainability of the breed. Although genetic diversity is currently preserved, indicators such as reduced effective population size, evidence of a founder effect, and higher inbreeding values in animals with complete pedigrees highlight the need for proactive genetic management.

The implementation of pedigree-based mating programs that integrate the inbreeding coefficient (F), average relatedness (AR), and the genetic conservation index (GCI) would allow for more efficient control of inbreeding while preserving founder diversity. In addition, balancing the reproductive contribution of breeding animals—particularly males—and promoting controlled gene flow among herds would help increase the effective population size and prevent genetic isolation.

Improving the completeness and accuracy of pedigree records is essential for effective genetic management, as incomplete data may lead to underestimation of inbreeding levels. Conservation strategies should therefore combine careful mating design with moderate selection, ensuring both genetic diversity and long-term productivity. Continuous genetic monitoring will be key to adapting these strategies over time.

### 4.4. Limitations of the Study

The analysis is based on existing records and therefore does not account for historical mating decisions or unrecorded breeding practices. These constraints may influence the estimation of genetic parameters and should be considered when applying the results to population management and conservation strategies. A limitation of this study is its reliance on genealogical data alone. Consequently, inbreeding and relatedness estimates derived from pedigree information may underestimate the true genetic relationships within the population, particularly when pedigree depth or integrity is limited. Future studies would benefit from incorporating molecular marker data to validate and complement pedigree-based estimates.

Despite these limitations, the present study provides the first comprehensive pedigree-based assessment of genetic diversity and population structure in the Sardo Negro breed across multiple herds. Although pedigree depth may lead to some underestimation of inbreeding parameters, the overall trends identified—particularly the decline in effective population size and the concentration of genetic contributions—offer relevant insights for genetic management. Integrating molecular marker data in future studies would allow validation of these findings and provide a more precise evaluation of genomic inbreeding and diversity. Together, pedigree and genomic approaches will be essential to support sustainable breeding strategies and long-term conservation of this locally adapted tropical breed.

## 5. Conclusions

This pedigree-based analysis indicates that the Sardo Negro cattle population currently maintains acceptable levels of inbreeding and overall genetic diversity despite its recent origin and structured breeding practices. However, the marked decline in effective population size in recent generations, together with the unequal use of breeding males and the concentration of genetic contributions in a limited number of ancestors, reveals a potential risk of future genetic erosion.

Although no recent bottleneck was detected, evidence of a founder effect and the discrepancy between global and complete-generation inbreeding estimates highlight the need for improved pedigree depth and data completeness to ensure accurate genetic evaluation.

The low genetic differentiation among herds reflects ongoing gene flow that contributes to maintaining diversity. Nevertheless, population growth alone does not ensure long-term genetic sustainability. Continuous monitoring of inbreeding and effective population size, together with balanced reproductive management, will be essential to preserve the breed’s genetic variability. This updated pedigree-based assessment complements recent genomic studies by providing demographic insights essential for managing inbreeding and maintaining diversity within the Mexican Sardo Negro breed.

## Figures and Tables

**Table 1:** Description of the population: Sardo Negro cattle in some subpopulations in Mexico.

Population	*n*	Percentage (%)
Total	8653	100
Females	4867	56
Males	3786	44
Dams	2367	49 ^1^
Sires	223	6 ^2^
Individuals without progeny	6063	70
Reference population	6840	79
Individuals with one known parent	67	0.8
Individuals with both parents unknown.	1763	20

^1^ Relative to the female population. ^2^ Relative to the male population.

**Table 2:** Generation interval and mean age at birth of offspring.

Pathways	GI	SE	AB	SE
Sire-Son	8.9	±0.5	8.1	±0.08
Sire-Daughter	7.6	±0.1	7.7	±0.07
Dam-Son	8.3	±0.4	8.6	±0.07
Dam-Daughter	8.0	±0.1	8.3	±0.07

GI: generation interval, AB: average age at birth of offspring, SE: standard error.

**Table 3:** Inbreeding and relatedness coefficient, percentage of inbred animals with their F estimations and effective population number by maximum generations traced in Sardo Negro cattle.

Generation ^1^	No. Animals	F %	Inbred Animals % (F %)	AR %	Ne
0	1748	0.0	---	0.1	–
1	1022	0.0	---	1.6	–
2	1634	1.5	9.6 (15.7)	2.2	33
3	846	3.7	28.4 (13.2)	3.6	22
4	1128	4.6	42.0 (11.1)	4.2	53
5	1137	4.8	51.1 (9.3)	3.6	387
6	574	4.7	57.0 (8.2)	3.7	–
7	360	5.2	81.4 (6.3)	3.6	124
8	196	4.8	85.2 (5.7)	3.4	–
9	8	3.0	75.0 (4.0)	3.6	–

^1^ Generations range from 1 recent to 9 distant. Negative estimates of Ne (–).

**Table 4:** Ancestors, probability of origin of diversity, Sardo Negro cattle.

Population, Metrics	*n*
Total	8653
Base population	1815
Effective number of founders	57.4
Number of animals in the reference population	6838
Number of founders	1187
Number of ancestors contributing to the reference population	1175
Effective number of ancestors in the reference population	32
Effective number of founders in the reference population	37
Number of ancestors explaining 50% of the ancestry	21

**Table 5:** Sires and calvings in their subpopulations or in separate herds.

Herd	Calvings	Own Sires % ^1^	Foreign Calvings % ^2^
1	2817	75.9	6.8
2	3872	38.1	8.2
3	176	68.6	0.6
4	430	68.6	1.3
5	1002	83.5	2.9
6	356	61.0	0.0

^1^ The remaining percentage corresponds to external sires. ^2^ The remaining percentage corresponds to offspring in the same subpopulation.

## Data Availability

The data sets presented in this article are not available due to privacy restrictions imposed by livestock breeders.
